# Training Frontline Providers in Basic Obstetric Ultrasound to Promote Healthy Pregnancy in Zimbabwe: Trainer and Trainee Experiences from a Qualitative Phenomenological Study

**DOI:** 10.3390/ijerph23081075

**Published:** 2026-08-19

**Authors:** Cladious Verenga, Shalote Chipamaunga-Bamu, Farai D. Madzimbamuto, Sunanda C. Ray

**Affiliations:** Faculty of Medicine and Health Sciences, University of Zimbabwe, Mount Pleasant, Harare P.O. Box MP 167, Zimbabwe; src@pcbillc.com (S.C.-B.); faraitose@hotmail.com (F.D.M.); acadreg@admin.uz.ac.zw (S.C.R.)

**Keywords:** obstetric ultrasound, point-of-care ultrasound, antenatal care, health workforce, interprofessional education, qualitative research, Zimbabwe

## Abstract

**Highlights:**

**Public health relevance—How does this work relate to a public health issue?**

- Limited access to trained obstetric ultrasound providers constrains basic antenatal risk detection and referral readiness in low-resource health systems.
- This study examines a Zimbabwean workforce-training model designed to extend focused basic obstetric ultrasound capacity to frontline antenatal care settings.

**Public health significance—Why is this work of significance to public health?**

- Participants experienced the six-week course as practical, confidence-building, and interprofessionally valuable, while emphasising the need for supervision and clear referral pathways.
- The findings clarify that basic obstetric ultrasound training should be framed as a supervised health-system capacity-building intervention, not as independent specialist sonographer training.

**Public health implications—What are the key implications or messages for practitioners, policy makers and/or researchers in public health?**

- Scale-up should be linked to equipment availability, mentorship, refresher training, competency assessment, image review, and specialist referral pathways.
- Future research should evaluate post-training practice, scan quality, completion of referrals, patient experience, and maternal–fetal outcomes.

**Abstract:**

Background: Limited access to trained providers remains a barrier to basic obstetric ultrasound use in low-resource antenatal care settings. This study aimed to explore trainer and trainee experiences of Zimbabwe’s six-week Basic Obstetric Ultrasound Short Course (BOUSC) and its perceived relevance to promoting healthy pregnancy through antenatal risk recognition and referral readiness. Methods: A qualitative study using transcendental phenomenology was conducted in the Fetal Medicine Units of Sally Mugabe Central Hospital and Parirenyatwa Group of Hospitals. Sixteen semi-structured interviews were conducted between September and October 2023 with eight trainers and eight trainees purposively selected from course records. Interviews were audio-recorded, transcribed verbatim, and analysed using Moustakas’ approach, including bracketing, horizontalisation, clustering of significant statements, and theme synthesis. Results: Thematic saturation was achieved. Four themes emerged: interactive and clinically situated teaching; confidence and basic skill development through supervised scanning; interprofessional learning that reduced hierarchy; and sustainability concerns related to equipment, mentorship, refresher training, and post-training practice. The most salient finding was that midwives trained as advanced obstetric sonographers were perceived as credible trainers for doctors and other cadres when operating within a supervised fetal medicine governance structure. Conclusions: Participants perceived the BOUSC as a practical and collaborative training model for basic obstetric ultrasound. The study does not demonstrate clinical outcome effects, but suggests that supervised basic ultrasound training may help promote healthy pregnancy when embedded within clear competency limits, quality assurance, and referral pathways.

## 1. Introduction

Promoting healthy pregnancy requires antenatal care systems that can identify risk early, support timely referral, and provide women with accurate information about pregnancy wellbeing. Obstetric ultrasound is one of the core technologies used to support these functions. The World Health Organization (WHO) recommends one ultrasound scan before 24 weeks of gestation to estimate gestational age, improve detection of fetal anomalies and multiple pregnancy, reduce induction of labour for post-term pregnancy, and improve women’s pregnancy experience [1,2]. International guidance also recognises the value of the 11–14-week scan when trained providers and health-system conditions make this feasible [3]. However, in low-resource settings, first-trimester access is often constrained by late booking, distance, equipment limitations and a shortage of trained providers. In Zimbabwe, the National Health Strategy 2021–2025 reports that only 29% of pregnant women booked early, before 16 weeks of gestation, according to MoHCC DHIS2 2019 data [4].

Against this background, the WHO recommendation for a scan before 24 weeks remains a pragmatic minimum target for many low-resource health systems, while earlier scanning should remain an aspiration as antenatal access improves. Basic obstetric ultrasound can support healthy pregnancy promotion by confirming viability, estimating gestational age, identifying multiple pregnancy, assessing fetal presentation, localising the placenta, assessing amniotic fluid, measuring basic fetal biometry, recognising selected red flags, and triggering referral. These functions are distinct from comprehensive anomaly screening, Doppler assessment, or specialist fetal medicine diagnosis, which require more advanced training and governance.

Despite its relevance, the benefits of obstetric ultrasound are not realised equitably. In many low- and middle-income countries (LMICs), access is limited by shortages of trained providers, concentration of sonography services in urban or referral hospitals, equipment limitations, maintenance challenges, and the absence of ultrasound training in routine pre-service curricula for many frontline cadres [5,6,7,8]. In sub-Saharan Africa, universal access to obstetric ultrasound remains a major implementation challenge, and the literature has highlighted task sharing, focused training, tele-support, and point-of-care models as strategies for expanding antenatal imaging capacity [5,9,10,11,12]. These models require careful attention to training quality, scope of practice, supervision, quality assurance, and referral pathways so that ultrasound access improves care rather than creating false reassurance or unsupported diagnoses.

Workforce training should therefore be understood as a public health and health-promotion intervention, not only as a health professions education activity. The International Society of Ultrasound in Obstetrics and Gynecology (ISUOG) has issued recommendations for basic training in obstetric and gynecological ultrasound, emphasising structured theoretical and practical training, supervision, and competence assessment [13]. More recent point-of-care ultrasound guidance also recognises that different healthcare professionals, including obstetricians, residents, midwives, and family physicians, may use focused ultrasound in defined clinical scenarios when training, governance, and referral mechanisms are appropriate [9,14].

In Zimbabwe, the Ministry of Health and Child Care encouraged expansion of obstetric ultrasound training following the adoption of WHO antenatal ultrasound guidance. The Fetal Medicine Units at Sally Mugabe Central Hospital and Parirenyatwa Group of Hospitals developed a six-week Basic Obstetric Ultrasound Short Course (BOUSC) combining theory, supervised scanning, specialist clinic exposure, practical assessment, and referral pathway orientation. The course was designed to address the shortage of providers capable of performing basic obstetric ultrasound and to support wider access to antenatal risk assessment.

The aim of this study was to explore the lived experiences and perceptions of trainers and trainees who participated in the BOUSC. The ultrasound training model may contribute to health promotion through workforce capacity-building, antenatal risk recognition, interprofessional collaboration, and referral readiness in a low-resource health system.

## 2. Materials and Methods

### 2.1. Study Design

A qualitative study using transcendental phenomenology was conducted to explore the essence of trainer and trainee experiences of the BOUSC. Transcendental phenomenology is a qualitative research approach, derived from Husserl’s phenomenology and operationalised for research by Moustakas, that seeks to describe the essential structure of a lived experience from participants’ descriptions while the researcher consciously brackets prior assumptions [15]. It was appropriate because the study sought to understand how participants experienced a specific educational and clinical training phenomenon rather than to measure competency outcomes or test a causal intervention. Reporting was guided by principles relevant to qualitative interview studies, including transparent description of setting, participants, data collection, analysis, and reflexivity [16].

### 2.2. Setting and Governance of Clinical Practice

The study was conducted in the Fetal Medicine Units of Sally Mugabe Central Hospital and Parirenyatwa Group of Hospitals in Harare, Zimbabwe. These units function as referral and training sites for fetal medicine and obstetric ultrasound. Training occurs within routine and specialist fetal medicine clinics, including clinics for multiple pregnancy, fetal defects, placenta disorders, and preterm surveillance. Within the units, the fetal medicine specialist has overall responsibility for clinical governance and final clinical decisions. A written care pathway guides the response to suspected abnormal findings. Trainees and basic scan providers are expected to recognise and document findings within their defined scope and refer suspected abnormalities to specialist clinics led by fetal medicine specialists and advanced obstetric sonographers trained in fetal medicine care pathways.

### 2.3. Description of the Basic Obstetric Ultrasound Short Course

The BOUSC is a six-week programme designed to equip trainees with competencies for focused basic obstetric ultrasound, not to train independent ultrasound technologists, comprehensive fetal anomaly screeners, or Doppler practitioners. The course combines approximately 10 h of theoretical teaching with supervised practical scanning throughout the six-week clinical attachment. This structure is consistent with other focused obstetric point-of-care ultrasound training models in African and low-resource settings, where short intensive teaching is combined with supervised practice, mentorship, or image review [10,11,12,17].

The theoretical component covers ultrasound safety, basic physics and knobology, image orientation, patient positioning, fetal lie and presentation, pregnancy dating, fetal viability, placental localisation, relationship of the placenta to the cervix, amniotic fluid assessment using the single deepest pool, basic fetal biometry including biparietal diameter/head circumference, abdominal circumference and femur length, growth assessment, report writing, recognition of findings outside the basic scope, and referral care pathways. Doppler assessment and comprehensive fetal anomaly screening are not competencies for independent practice in this course; where advanced topics are mentioned, this is for awareness, red-flag recognition, and timely referral rather than independent diagnostic certification.

The practical component is delivered in fetal medicine clinics by midwives who have undergone two years of advanced obstetric ultrasound training under the supervision of Fetal Medicine Foundation Zimbabwe and Fetal Medicine Foundation UK and are certified as advanced obstetric sonographers. Trainees observe expert scanning, practise under direct hands-on guidance, progress to supervised independent scanning, and receive feedback on image planes, caliper placement, interpretation, documentation, and referral. All trainee scans are double-checked by trainers. Suspected abnormal findings are referred through the unit’s specialist care pathway for review by the fetal medicine specialist and the advanced fetal medicine team.

The exit assessment uses a competency checklist rather than a numerical score. Competencies include patient positioning, image orientation, fetal lie/presentation, zoom, basic biometry planes and caliper placement, placental localisation, relationship of the placenta to the cervix, deepest vertical pool, fetal activity, and first-trimester viability where applicable. Trainers classify performance as Very Good, Good, Average, or Insufficient. A certificate of competency is issued only when the trainer judges that the trainee can perform the defined basic scan tasks safely within the referral pathway (Supplementary S2).

### 2.4. Participants and Sampling

Participants were identified from Fetal Medicine Unit training records. The potential trainer population consisted of ten midwives trained in advanced obstetric ultrasound; eight trainers were available and participated. The potential trainee population consisted of 20 health workers who had attended the six-week course; 15 were contactable, and eight were purposively selected to provide variation by profession, age, and experience. The purpose of sampling was not numerical representativeness but depth and variation of experience, consistent with qualitative phenomenological research. Interviews stopped after the eighth trainee interview because data saturation had been reached; no substantially new codes or themes were emerging, and responses were becoming repetitive across participant groups. The final sample consisted of 16 participants: eight trainers and eight trainees.

### 2.5. Data Collection

Semi-structured interviews were conducted between 14 September and 1 October 2023. Trainers based at the study sites were interviewed in person, while trainees who were not available at the study sites were interviewed by telephone. The interview guide explored participants’ professional background, how they came to participate in the course, descriptions of their course experience, emotional responses to the course, views on interprofessional teaching and learning, expectations of the course, barriers and facilitators during training, and suggested improvements (Supplementary S1). With permission, interviews were audio-recorded on a mobile phone and transcribed manually, verbatim. Field notes and analytic memos were used during familiarisation and interpretation.

### 2.6. Data Analysis

Data analysis followed Moustakas’ transcendental phenomenological approach [15]. Transcripts were read and re-read while listening to recordings to enhance familiarisation. Significant statements related to course experiences were identified through horizontalisation, where each relevant statement was initially treated as having equal value. Statements were then clustered into codes and categories, followed by development of textural descriptions of what participants experienced and structural descriptions of how the training context shaped these experiences. Ten initial themes were consolidated into four final themes through constant comparison with the original transcripts, recordings, analytic memos, and research questions. Coding and theme development were manual (Supplementary S3).

### 2.7. Trustworthiness and Reflexivity

Credibility was strengthened through purposive sampling across trainer and trainee groups, triangulation of trainer and trainee perspectives, verbatim transcription, repeated reading of transcripts, and use of participant quotations to support themes. The interview guide was piloted, and the research tools were reviewed by academic supervisors. Reflexivity was addressed through bracketing: the researcher piloted the interview process as a participant with a research assistant conducting the interview, allowing prior assumptions and biases to be surfaced before data collection. Anonymity was maintained using participant numbers. The risk of selection bias was acknowledged because the trainees who were contactable and available may have been more motivated or more positively disposed toward the course.

### 2.8. Ethical Considerations

The study was conducted in accordance with ethical principles for research involving human participants. Written informed consent was obtained from all participants; telephone participants received consent materials electronically. Participants were informed about recording, confidentiality, anonymity, voluntary participation, and the academic purpose of the study. Ethical approval was granted by the Joint Research Ethics Committee for the University of Zimbabwe Faculty of Medicine and Health Sciences and Parirenyatwa Group of Hospitals (JREC/305/2023; approval date 13 September 2023; expiry date 12 September 2024). Institutional permission was also obtained from Sally Mugabe Central Hospital; the institutional permission letter did not provide a separate approval number.

### 2.9. Use of Generative Artificial Intelligence in Manuscript Preparation

During preparation and revision of this manuscript, the authors used ChatGPT (GPT-5.5; OpenAI, San Francisco, CA, USA) to support language editing, and strengthening of the public health and health-promotion framing. The tool was not used for data collection, participant recruitment, coding, theme generation, or primary data analysis. The authors reviewed and edited all AI-assisted output and take full responsibility for the content of this publication.

## 3. Results

### 3.1. Participant Characteristics

Sixteen participants were interviewed: eight trainers and eight trainees. Participants were aged 23–44 years. Trainers were all midwives who had been trained and certified as advanced obstetric sonographers. Trainees included midwives, medical doctors, a student radiographer, a student sonographer, and a specialist obstetrician and gynaecologist, with 1–12 years of professional experience. The sample reflected the interprofessional nature of the BOUSC (Table 1).

### 3.2. Theme 1: Interactive, Practical, and Clinically Situated Teaching and Learning

Participants described the course as clinically meaningful because it took place in real fetal medicine clinics rather than in a purely classroom-based environment. Although participants used positive descriptors such as ‘good experience’, ‘wonderful’, ‘enjoyable’, ‘beneficial’, and ‘eye opening’, their accounts also described concrete learning processes: demonstration by trainers, hands-on repetition, immediate correction of image planes and caliper placement, access to senior support, and exposure to a range of antenatal cases. A trainee summarised the design as follows: ‘The training methods were good as it combined both theory and practice skills assessment at the end’ (Participant 10).

The main facilitator was the integration of teaching with daily clinical work. Trainees described trainers as accessible and supportive, while trainers noted the need for patience because participants entered the course with different professional backgrounds and baseline ultrasound exposure. The main barriers were uneven access to machines, variable pace of learning, and the need for more structured theory reinforcement. These findings suggest that participants’ accounts went beyond general expressions of satisfaction; they described a supervised clinical learning process that connected basic scan acquisition with documentation and referral.

### 3.3. Theme 2: Confidence and Basic Skill Development Through Supervised Scanning

Trainees reported that supervised scanning improved their confidence, systematic technique, and professional approach to basic obstetric ultrasound. One trainee described ‘a lot of time to scan independently but also there was senior support available’ (Participant 15). Another stated that they ‘got to learn to scan in a proper professional way’ (Participant 14). Participants valued exposure to routine and specialist clinics because this helped them understand the boundary between basic ultrasound tasks and abnormalities requiring referral.

Participants linked the basic skills to potential clinical usefulness. One participant described the benefit to pregnant women as being able to ‘diagnose abnormalities early’ (Participant 12), while another stated that the course could help providers ‘manage patients in a proper way’ (Participant 14). In the revised framing, these statements are interpreted cautiously as participant perceptions of potential value rather than evidence of measured diagnostic accuracy or improved outcomes.

### 3.4. Theme 3: Interprofessional Learning Reduced Hierarchical Barriers

Interprofessional learning was initially associated with some anxiety. Some doctors and radiography trainees were uncertain about being trained by midwife sonographers, while some midwife trainers were concerned about teaching colleagues who traditionally occupied higher positions within professional hierarchies. A trainee reflected: ‘I was quite worried that it would be difficult’ (Participant 13). Another initially ‘wasn’t confident enough at the beginning being trained by midwives’ but later realised ‘how good and knowledgeable they are’ (Participant 10).

This interprofessional dynamic should be understood within the unit’s governance structure. The midwife trainers were not basic-level trainees; they were midwives who had undergone two years of advanced obstetric ultrasound training and certification under specialist supervision. The fetal medicine specialist retained overall clinical responsibility, and suspected abnormal findings were referred through the specialist pathway. Within this structure, participants reported that shared learning created a common ultrasound language and increased respect for midwife sonographers. One participant captured this shift by saying, ‘if the doctor gets to see a midwife sonographer they will not run away because we will be talking the same language’ (Participant 14).

### 3.5. Theme 4: Sustainability, Continuing Practice, and Future Professional Development

Although participants perceived the course positively, they raised sustainability concerns. These included the need for more ultrasound machines, opportunities for continued scanning after training, refresher courses, post-training supervision, mentorship, and structured follow-up. Some participants expressed frustration that trainees could have difficulty continuing to use their new skills after returning to their workplaces, especially where equipment access or local supervision was limited.

Trainees generally considered the six-week duration adequate for the defined basic scope, but some requested more theory teaching and more formal theory assessment. Participants suggested that a longer or diploma-level programme could support career progression and professional recognition. The sustainability theme therefore extended beyond individual learning: participants saw continued mentorship, equipment availability, quality assurance, and integration into national or university curricula as necessary for long-term health-system impact (Table 2).

## 4. Discussion

This study explored trainer and trainee experiences of Zimbabwe’s BOUSC and interpreted these findings within a health-promotion and public health framework. The principal finding is that participants experienced the course as practical, confidence-building, and interprofessionally valuable, while also identifying sustainability needs that must be addressed before scale-up. The most distinctive finding was the interprofessional supervisory model: midwives certified as advanced obstetric sonographers were perceived as credible trainers for doctors and other cadres when the training occurred within a specialist-led fetal medicine unit, with double-checking and clear referral pathways. This approach aligns with Kolb’s experiential learning model [18].

The study should not be interpreted as evidence that BOUSC directly reduced maternal or perinatal morbidity or mortality, or as proof of diagnostic accuracy. Rather, the findings suggest that participants perceived the course as improving readiness to perform focused basic obstetric ultrasound tasks and to refer suspected abnormalities. This interpretation is consistent with the qualitative design, in which the purpose was to describe lived experiences rather than to measure clinical outcomes.

The health-promotion relevance of the BOUSC lies in its focus on capacity-building for antenatal risk recognition. WHO recommends an ultrasound scan before 24 weeks of gestation because of its value for dating, multiple pregnancy detection, fetal anomaly detection, reduction in post-term induction, and improved pregnancy experience [1,2]. ISUOG guidance supports the value of 11–14-week ultrasound when feasible [3], but Zimbabwean national data show that early booking before 16 weeks remains low at 29% [4]. A realistic ultrasound strategy in such contexts must therefore balance the ideal of early first-trimester scanning with pragmatic efforts to expand access before 24 weeks and through later antenatal care contacts.

The findings are consistent with literature from sub-Saharan Africa describing obstetric ultrasound access as constrained by shortages of trained providers and inequitable service distribution [5,6,7,8]. Similar studies in Uganda and Kenya report that midwives, nurses, and rural healthcare providers can be trained in focused obstetric point-of-care ultrasound when training is linked to defined applications and implementation support [10,11]. The Tanzania/Zanzibar and Rwanda experiences also demonstrate that focused ultrasound programmes in low-resource settings often combine short theoretical components with extended practical exposure, supervision, tele-review, or longitudinal mentorship [12,17]. This literature supports the clarification that a six-week course is not equivalent to full sonographer training; it is a focused basic course for defined tasks within a referral system.

A clear definition of the course scope is essential. Basic obstetric ultrasound in this programme refers to presentation, viability, placental localisation, amniotic fluid assessment, basic biometry, growth assessment, documentation, and referral. It does not certify independent competence in Doppler, comprehensive anomaly screening, or advanced fetal medicine diagnosis. This distinction is essential for patient safety, regulatory clarity, and medicolegal governance. ISUOG basic training and point-of-care ultrasound guidance both emphasise that training should be matched to intended scope of practice and that clinical governance is required when ultrasound is performed by different professional cadres [9,13,14].

The interprofessional learning findings are important for implementation. Participants reported that the course reduced hierarchy between midwives, doctors, radiographers, and sonographers. The literature on interprofessional education suggests that shared learning can improve collaboration and selected healthcare processes, although effects vary by context and intervention design [19]. In this study, shared ultrasound learning appeared to create a common clinical vocabulary and greater respect for midwife sonographers. The governance model remains central: midwife trainers provided advanced sonographic teaching, but final clinical accountability for abnormal findings rested with the fetal medicine specialist, supported by the specialist clinic pathway.

Participants’ sustainability concerns are central to policy and implementation. Training alone is insufficient if graduates return to facilities without machines, mentorship, quality assurance, or opportunities to practise. Participants’ requests for refresher training, follow-up supervision, more equipment, and diploma-level progression are consistent with the broader literature on task sharing and ultrasound training in LMICs [7,8,10,11,12]. Unsupported expansion could result in skill decay, inconsistent scan quality, and weak referral linkages.

Emerging approaches such as tele-ultrasound, remote image review, handheld ultrasound, and artificial intelligence-assisted acquisition may complement human capacity-building but should not replace training and governance. Studies from resource-limited settings suggest that handheld and AI-supported ultrasound approaches may support selected tasks such as urgent obstetric assessment, gestational age estimation, fetal malpresentation, and quality assurance [20,21,22,23]. For Zimbabwe, these tools could be considered as adjuncts to a supervised training pathway, especially for remote areas, but local validation, data governance, referral capacity, and safety monitoring would be required before routine scale-up.

For Zimbabwe and similar settings, the policy implication is that BOUSC-type training should be integrated into a tiered maternal–fetal care pathway. Such a pathway would define basic scan competencies, referral criteria, trainer requirements, supervision models, image review processes, equipment maintenance plans, and data systems for monitoring scan quality and clinical follow-up. Integration into Ministry of Health and Child Care programmes and University of Zimbabwe pre-service or postgraduate curricula could support sustainability, while fetal medicine units could function as mentorship hubs for district and provincial providers.

This study has several strengths. It included both trainers and trainees, captured interprofessional perspectives, used a phenomenological design appropriate for lived experience, and drew on participants involved in a real training programme embedded within public referral hospitals. The study has limitations. It was conducted in two linked referral hospital training sites, included a small qualitative sample, and relied on self-reported perceptions rather than observed competence, diagnostic accuracy, post-training practice, referral timeliness, or maternal–fetal outcomes. Selection bias is possible because participants were drawn from those contactable and available for interview, and social desirability bias may have influenced responses despite bracketing and anonymity. Future studies should use mixed methods to assess competency retention, scan quality, patient experience, referral outcomes, and the effect of mentorship and equipment availability on sustained practice.

## 5. Conclusions

The Basic Obstetric Ultrasound Short Course was experienced by trainers and trainees as practical, confidence-building, and supportive of interprofessional collaboration. Participants perceived the course as relevant to basic antenatal risk recognition and referral readiness, but they also highlighted that sustained value depends on equipment access, refresher training, mentorship, quality assurance, defined scope of practice, and integration into broader antenatal care and referral systems.

The study contributes to the health-promotion literature by framing basic obstetric ultrasound training as a supervised health-system capacity-building intervention that supports healthy pregnancy in a low-resource setting. It does not demonstrate clinical outcome effects. The findings support further development and evaluation of structured, supervised ultrasound training models in Zimbabwe and similar contexts, with future research measuring competence, practice sustainability, referral quality, patient experience, and maternal–fetal outcomes.

## Figures and Tables

**Table 1:** Participant characteristics.

Group	*n*	Professional Profile	Age and Experience
Trainers	8	Midwives certified as advanced obstetric sonographers after extended advanced ultrasound training	Age 35–44 years; experience 1–7 years
Trainees	8	Midwives (2); medical doctors (3); student radiographer (1); student sonographer (1); specialist obstetrician and gynaecologist (1)	Age 23–43 years; experience 1–12 years
Total	16	Interprofessional sample of trainers and trainees	Age 23–44 years; experience 1–12 years

**Table 2:** Final themes and health-promotion interpretation.

Theme	Core Finding	Health-Promotion Interpretation
Interactive and practical teaching	Participants valued demonstration, hands-on practice, feedback, accessible trainers, and real clinical exposure.	Learning was aligned with basic antenatal care tasks and may support appropriate use of ultrasound within a defined scope.
Confidence and basic skill development	Supervised scanning supported confidence, systematic technique, and professional reporting.	Confidence and foundational skills are prerequisites for local risk recognition and referral readiness.
Interprofessional learning	Midwives certified as advanced obstetric sonographers trained doctors and other cadres within a specialist-governed unit.	Shared ultrasound language may improve team communication, respect, and referral practice.
Sustainability concerns	Participants requested equipment, mentorship, refresher courses, supervision, and progression pathways.	Training must be embedded in health-system support to maintain quality and avoid skill decay.

## Data Availability

The qualitative interview data are not publicly available because they contain information that could compromise participant confidentiality. De-identified excerpts supporting the findings are included in the manuscript. Further access may be considered by the corresponding author subject to ethical approval and institutional permission.

## Supplementary Materials

The following supporting information can be downloaded at https://www.mdpi.com/article/10.3390/ijerph23081075/s1, Supplementary S1: Semi-structured interview guide used for trainers and trainees. Supplementary S2: BOUSC trainee assessment form. Supplementary S3: Coding process summary showing initial codes and final theme development.
