# Supplementary material for: Training Frontline Providers in Basic Obstetric Ultrasound to Promote Healthy Pregnancy in Zimbabwe: Trainer and Trainee Experiences from a Qualitative Phenomenological Study
Source: Int J Environ Res Public Health. 2026 Aug 19;23(8):1075. doi: 10.3390/ijerph23081075 (PMC13513090; doi:10.3390/ijerph23081075)
Supplement: Supplementary file 1 [file ijerph-23-01075-s001.zip › ijerph-4386922-supplementary.pdf]

Supplementary Materials

Supplementary S1. Semi-Structured Interview Guide

1. May you please tell us about yourself and how you came about to be in the course?
2. Probe: age, gender, occupation, years of experience in the profession, and number trained (for trainers).
3. How would you describe your experience of the course?
4. Probe: course content, teaching methods, and duration.
5. Do these experiences reflect your overall feelings of the course?
6. Probe: Can you expand/explain? What words best describe your feelings about the course?
7. This course is interprofessional. Midwives, radiographers, and doctors teach and learn together. Can you describe how you felt about that?
8. What expectations did you have of the course? Did you feel the course met your expectations? Were there parts of the course you felt could have been changed (longer, shorter, removed, etc.)?

Supplementary S2. BOUSC Trainee Assessment Form

Assessment scale: Well acquired; acquired; acquired but to be consolidated; yet to learn.

**Competency area**	**Assessment items**
Knobology and basics	Positioning of patient for examination; image orientation; fetal position in relation to mother; situs; zoom
Biometry	BPD-HC plane and caliper position; AC plane and caliper position; FL plane and caliper position
Placenta and cord	Placenta localisation and relationship with cervix
Amniotic fluid	Deepest amniotic fluid pool
Fetal activity	Fetal movements: respiratory movements, flexion, extension
First trimester	Fetal viability
Final evaluation	Very good; good; average; insufficient; trainer and trainee signatures

Supplementary S3. Coding Process Summary

- Teaching methods: practical teaching and accessible instructors.
- Course duration: adequate for many participants, but some suggested a longer or diploma-level course.
- Exposure and training environment: good experience, conducive environment, diverse cases, senior support.
- Theory learning: request for more theory content and more theory grilling.
- Enjoyment and satisfaction: enjoyable experience and satisfying course.
- Confidence and skill improvement: increased professional skills and gained confidence.
- Importance of the course: beneficial to patients and healthcare professionals, important for managing pregnancies.
- Structure and content: unique and well-structured course with above-average content.
- Interprofessional learning: positive experience; integration helped learning.
- Expectations: course mostly met expectations; some suggestions for improvement.
